# Dihydromyricetin Induces Apoptosis and Reverses Drug Resistance in Ovarian Cancer Cells by p53-mediated Downregulation of Survivin

**DOI:** 10.1038/srep46060

**Published:** 2017-04-24

**Authors:** Yingqi Xu, Shengpeng Wang, Hon Fai Chan, Huaiwu Lu, Zhongqiu Lin, Chengwei He, Meiwan Chen

**Affiliations:** 1 State Key Laboratory of Quality Research in Chinese Medicine, Institute of Chinese Medical Sciences, University of Macau, Macau, China; 2 Department of Biomedical Engineering, Columbia University, New York, NY 10027, USA; 3 Department of Gynecologic Oncology, Sun Yat-sen Memorial Hospital, Sun Yat-sen University, Guangzhou, Guangdong, China

## Abstract

Ovarian cancer is one of the leading causes of death in gynecological malignancies, and the resistance to chemotherapeutic agents remains a major challenge to successful ovarian cancer chemotherapy. Dihydromyricetin (DHM), a natural flavonoid derived from *Ampeopsis Grossdentata*, has been widely applied in food industry and medicine for a long time. However, little is known about the effects of DHM on ovarian cancer and the underlying mechanisms. In this study, we demonstrated that DHM could effectively inhibit the proliferation of ovarian cancer cells and induce cell apoptosis. Survivin, an inhibitor of apoptosis (IAPs) family member, exhibited a decreased expression level after DHM treatment, which may be attributed to the activation of p53. Moreover, DHM markedly sensitized paclitaxel (PTX) and doxorubicin (DOX) resistant ovarian cancer cells to PTX and DOX by inhibiting survivin expression. Collectively, our findings highlight a previously undiscovered effect of DHM, which induces apoptosis and reverses multi-drug resistance against ovarian cancer cells through downregulation of survivin.

Ovarian cancer is one of the most lethal gynecological malignancies in the USA and an estimated 14,180 deaths are expected in 2015^1^. Ovarian cancer is typically asymptomatic in its early stages, which decreases the chances of early diagnosis^2^. An ovarian cancer patient who is diagnosed at early stages has a survival rate of 99% throughout his/her lifetime. However, 75% of ovarian cancer patients who are suffering from peritoneal metastasis at the time of diagnosis have a five-year survival rate of less than 30%^3^. Treatments generally consist of conventional cytotoxic chemotherapy and surgical therapy^4^. Despite successful initial treatment, as many as 70% of ovarian cancer patients would develop advanced-staged disease and resistance to treatment, depriving long-term benefit from treatment^5^. This observation highlights the importance of better understanding the mechanism of therapy and devising more effective strategies to conquer therapeutic resistance.

Upregulation of inhibitors of apoptosis proteins (IAPs) expression has been associated with drug resistance in various cancers^6^. Survivin, a nodal protein of IAPs family, is abundantly expressed in 60 cancer cell lines and in most common human neoplasms^7^,^8^. Survivin is a potential molecular target in cancer therapy because of its important roles in inhibiting apoptosis, enhancing proliferation and promoting angiogenesis. Overexpression of survivin protects cells from various apoptotic stimuli such as chemotherapeutic agents, whereas reducing survivin expression or function can result in enhanced sensitivity to exposure to apoptotic stimuli or the spontaneous apoptosis of cancer cells^9^. Although the regulation of survivin is not completely understood, it has been reported that the retinoblastoma protein and p53 are required for survivin gene repression^10^. Furthermore, some transcription factors, such as nuclear factor kappa B (NF-κB), can promote survivin gene transcription^11^. Moreover, survivin is overexpressed approximately 40-fold in tumor tissues and renders cancer cells resistant to radiotherapy and chemotherapy. Therefore, identifying survivin inhibitors represents an important step of effective cancer treatment.

Dihydromyricetin (DHM) (Fig. 1A) is a flavonoid that can be isolated from the stems and leaves of *Ampelopsis grossedentata*^12^. It is associated with multiple pharmacological benefits by performing anti-inflammatory, antioxidant, antibacterial, antihypertensive and antithrombotic activities^13^,^14^,^15^. Moreover, previous studies have demonstrated its potent antitumor activity against a broad range of cancers, including breast cancer, liver cancer, colon cancer, and lung cancer^16^,^17^,^18^,^19^,^20^. DHM can inhibit cancer cell proliferation and can induce cell cycle arrest and apoptosis. It is also known to sensitize cancer cells to chemotherapeutic drugs^21^,^22^,^23^,^24^. However, little is known about its effects on ovarian cancer, and the underlying mechanisms of its anticancer effects still require further investigation. In this study, we aimed to investigate the therapeutic potential of DHM on ovarian cancer and explore its underlying mechanisms of action. Furthermore, the effects of DHM combined with chemotherapeutic agents against resistant ovarian cancer cells were evaluated.

## Results

### DHM inhibits proliferation and induced G0/G1 and S phase arrest in ovarian cancer cells

**Figure 1:**
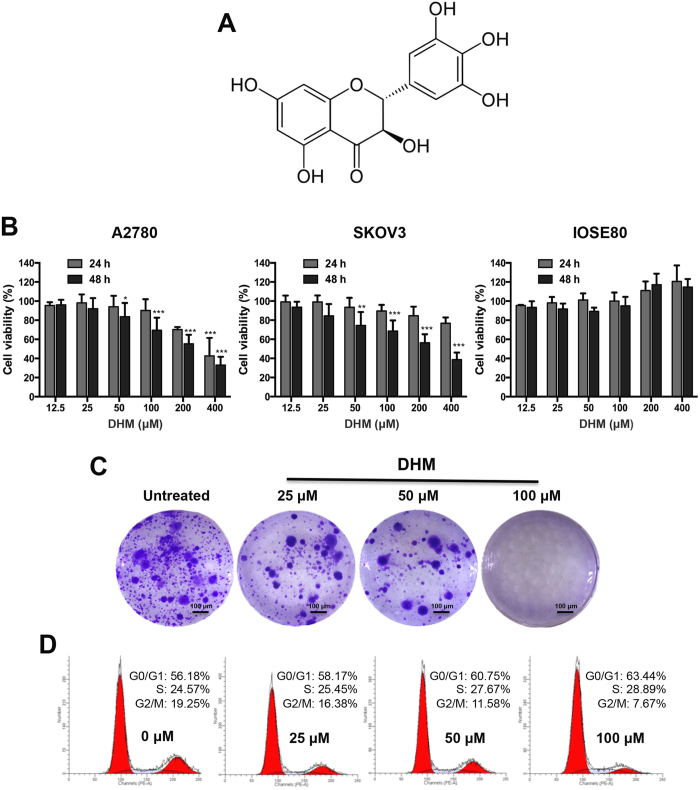
The effects of DHM on ovarian cancer cell proliferation. (**A**) Chemical structure of DHM. (**B**) A2780 cells, SKOV3 cells and ISOE80 cells were exposed to different concentrations of DHM for 24 and 48 h. Cell viability was determined by a MTT assay. (**C**) Colony formation assays of A2780 cells after treatment with different concentrations of DHM (25, 50, 100 μM). (**D**) The effects of different concentrations of DHM (25, 50, 100 μM) on the cell cycle distribution of A2780 cells. Data are expressed as mean ± SD for three independent experiments. *p < 0.05, **p < 0.01, ***p < 0.001.

The *in vitro* anti-proliferation effect of DHM was assessed in A2780 and SKOV3 ovarian cancer cells and IOSE80 human ovarian epithelial cells. The cells were seeded in 96-well plates at a density of 5 × 10^3^ cells/well 24 h prior to DHM exposure. The cells were treated with various DHM concentrations (12.5, 25, 50, 100, 200 and 400 μM). MTT assay was conducted to detect the cell viability after treatment with different concentrations of DHM for 24 and 48 h. As shown in Fig. 1B, DHM inhibited cell proliferation in A2780 and SKOV3 ovarian cancer cells in a concentration- and time-dependent manner. In p53 positive A2780 cells, the IC_50_ value was 336.0 μM after DHM treatment for 24 h. However, in p53 null SKOV3 cells, the IC_50_ value was 845.9 μM, which was 2.5-fold higher than that in A2780 cells. We also tested whether DHM was cytotoxic to normal ovarian cells. Interestingly, no significant cytotoxicity was observed in human ovarian surface epithelial IOSE80 cells after DHM treatment. Next, to confirm the suppressive effects of DHM on ovarian cancer cell proliferation, we performed colony formation assay on A2780 cells. The cells were exposed to 25, 50 and 100 μM of DHM for 48 h, and were continued to be cultured for 2 weeks in fresh medium until colonies formed. Consistent with the results of the MTT assay, the colony formation capacity was observably reduced with increasing concentrations of DHM, demonstrating that cell proliferation was suppressed by DHM (Fig. 1C).

Previous studies have shown that DHM can induce cell cycle arrest in various types of cancer cells^24^,^25^. In this study, the cell cycle progression was examined on A2780 cells by flow cytometry. The cells were treated with different concentrations of DHM (0, 25, 50, 100 μM) for 24 h after starvation. As shown in Fig. 1D, DHM specifically arrested A2780 cells at the G0/G1 and S phase in a concentration-dependent manner. Specifically, after exposure to 100 μM of DHM for 24 h, the number of cells in G0/G1 increased from 56.18% to 63.44%, similar to the number observed in the S phase, whereas the percentage of cells in the G2/M phase decreased from 19.25% to 7.67%. The data displayed the significant cell cycle arrest effects of DHM on ovarian cancer cells at G0/G1 and S phase in a concentration-dependent manner.

### DHM induces cell apoptosis and activates the apoptosis-related signaling pathway

**Figure 2:**
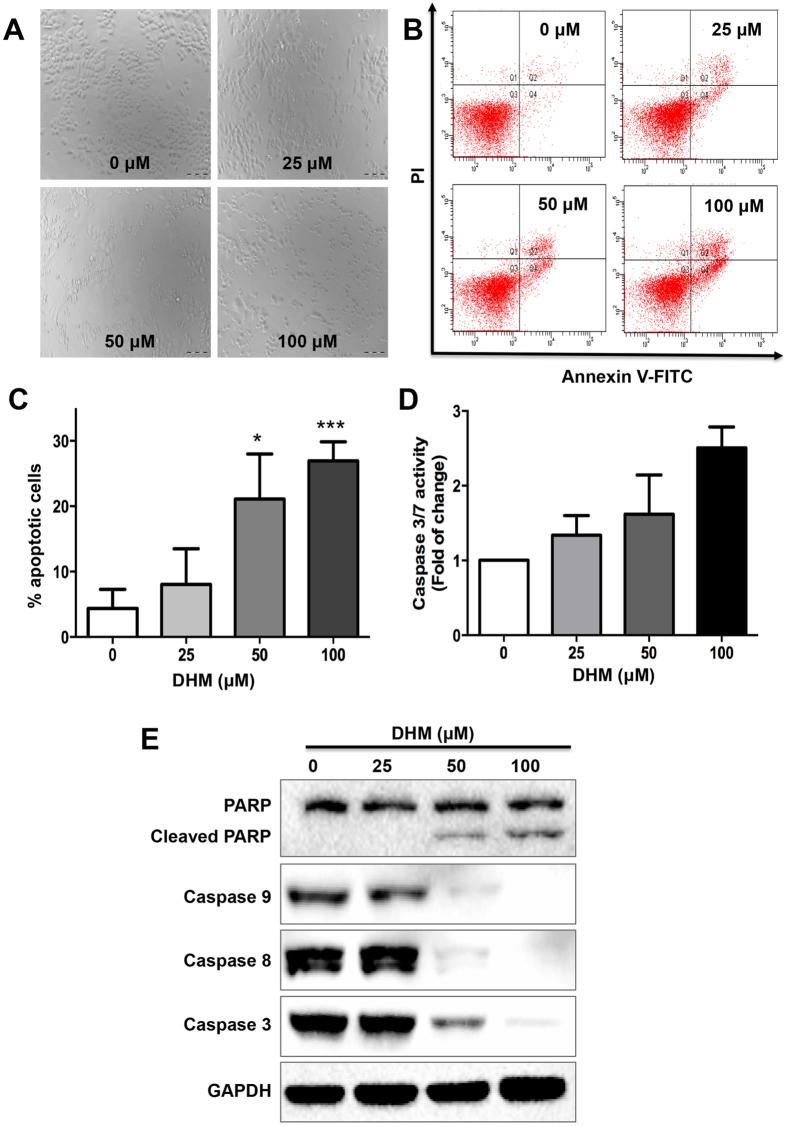
Effects of DHM on cell apoptosis. (**A**) Cell morphologies of A2780 cells treated with DHM (25, 50, 100 μM) for 48 h. (**B**) A2780 cells were treated with different concentrations of DHM (25, 50, 100 μM) for 48 h and analyzed with flow cytometry after Annexin V-FITC/PI staining. Annexin-V-FITC^−^/PI^−^ populations in Quadrant 3 were non-apoptotic cells, while Annexin-V-FITC^+^/PI^−^cells in Quadrant 4 and Annexin-V-FITC^+^/PI^+^ cells in Quadrant 2 were considered late apoptotic cells, respectively. (**C**) The apoptotic ratio of A2780 cells was calculated. (**D**) The Caspase 3/7 activity was determined after exposure to DHM for 24 h. (**E**) Expression of activated PARP, caspase 8 and caspase 9 were determined by Western blotting. Results were obtained based on two or three separate experiments. Data were expressed as mean ± SD. *p < 0.05, ***p < 0.001.

To explore whether the deregulation of the cell cycle was correlated with the induction of apoptosis, cell morphology was observed and Annexin V-FITC/PI staining was performed after DHM treatment for 48 h. As shown in Fig. 2A, while the untreated cells were rounded, cells became condensed and cell population showed dramatic depletions after DHM treatment. Moreover, A2780 cells treated with different concentrations of DHM for 48 h displayed significant levels of apoptosis in a concentration-dependent manner (Fig. 2B). The apoptotic rates of the A2780 cells in the presence of 25, 50 and 100 μM of DHM for 48 h were 12.1, 21.1, and 26.9%, respectively (Fig. 2C). The results confirmed that DHM specifically targeted p53 positive A2780 cells and promoted cell apoptosis, which were consistent with the results of MTT assay and cell cycle study.

We further confirmed this result by evaluating the caspase 3/7 activity using the Caspase3/7-Glo Assay Kit (Promega, Madison, USA) As expected, the apoptotic rates of the DHM-treated were 34.0% (25 μM), 61.9% (50 μM) and 150.7% (100 μM), respectively, which were higher than that of the control group (Fig. 2D), suggesting that an enhanced and concentration-dependent caspase 3/7 activity was induced by DHM. Next, we detected the expression of apoptosis-related protein poly ADR-ribose polymerase (PARP), caspase 8 and caspase 9 after DHM stimulation using a Western blotting detection kit. Of note, DHM treatment increased the expression of cleaved PARP and decreased the expression of caspase 3, 8 and 9 in a concentration-dependent fashion (Fig. 2E), demonstrating that PARP function was impaired via enhanced splicing, which caused cancer cell apoptosis.

### DHM induces apoptosis by downregulating the expression of survivin

**Figure 3:**
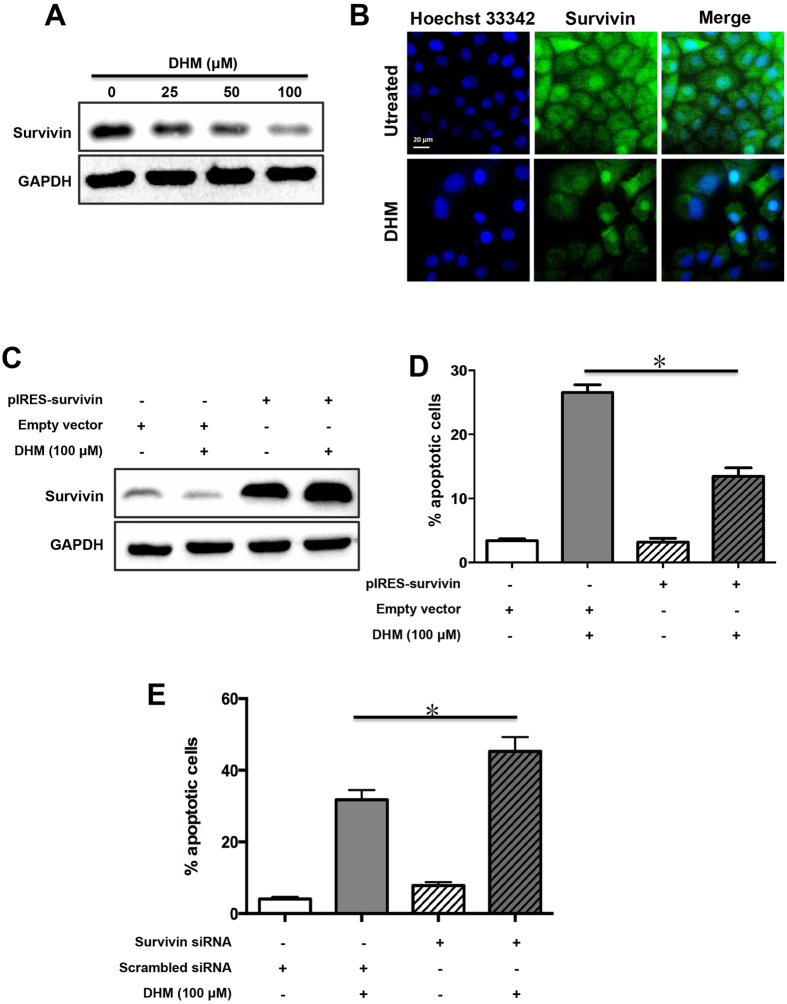
Effects of DHM on survivin expression in A2780 cells. (**A**) A2780 cells were treated with different concentrations of DHM for 48 h and the expression of survivin was evaluated using Western blotting. (**B**) Cells were treated with DHM for 48 h and the expression of survivin was observed using a fluorescent microscope. A2780 cells were transfected with pIRES-survivin vector following treatment with 100 μΜ of DHM for 48 h. The expression of survivin was assessed by Western blotting (**C**) and apoptosis rates were determined using flow cytometry (**D**). (**E**) A2780 cells were transfected with survivin siRNA and treated with 100 μM of DHM for 48 h. Apoptosis rates were determined using flow cytometry after Annexin V-FITC/PI staining. *p < 0.05.

Survivin, as an anti-apoptotic gene, was shown to be overexpressed in ovarian cancer^26^. Intrigued by the apoptotic effect of DHM induced in A2780 cells, we therefore sought to explore the effects of DHM on the expression of survivin. Cells were treated with DHM for 48 h, and the expression of survivin was examined by an immunoblot assay. As shown in Fig. 3A, compared with control cells, DHM downregulated the expression of survivin in A2780 cells in a concentration-dependent manner. This finding was further evidenced in the immunofluorescence images (Fig. 3B).

### Overexpression of survivin attenuates DHM-mediated apoptosis

To confirm whether DHM triggered apoptosis by decreasing the survivin expression, we performed survivin transfection taking advantage of the plasmid pIRES-survivin we constructed based on the previously reported method^27^. pIRES vector was adopted as the control. The results of transfection were confirmed with Western blotting analysis and flow cytometry. As shown in Fig. 3C, cells transfected with pIRES-survivin showed a higher expression level of survivin with or without DHM treatment. In contrast, in the empty vector groups, survivin was present at lower levels under the same condition. The results of Annexin V-FITC/PI dual staining showed that cells transfected with pIRES-survivin plasmid had a decreased apoptotic rate compared to that with empty plasmid when treated with 100 μM of DHM (Fig. 3D). On the contrary, the apoptotic rate of cells transfected with survivin siRNA reached 45.3% after DHM treatment for 48 h, 13.5% higher than that with scrambled siRNA due to survivin was knocked down by survivin siRNA(Fig. 3E).

### Activation of p53 was critical for DHM-induced apoptosis

**Figure 4:**
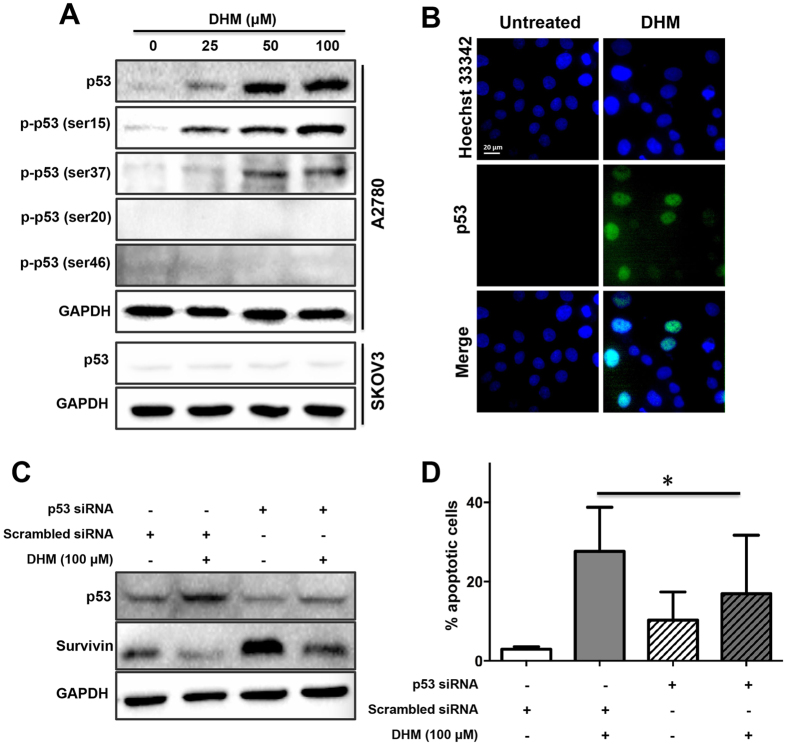
The role of p53 in DHM-induced cell apoptosis and downregulation of survivin. A2780 and SKOV3 cells were treated with different concentrations of DHM for 48 h, respectively, and the expression of p53 was evaluated using Western blotting (**A**) and immunofluorescence (**B**). (**C**) Cells were transfected with p53 siRNA following treatment with 100 μΜ of DHM for 48 h, and the expression levels of p53 and survivin were assessed by Western blotting analysis. (**D**) The cell apoptosis rates were determined using flow cytometry. *p < 0.05.

It was documented that p53 and survivin could together modulate cell apoptosis^28^,^29^. As the above data indicated, DHM triggered increased cell apoptosis in a concentration-dependent manner. Accordingly, we sought to verify the effects of p53 on DHM-mediated apoptosis using Western Blotting. As shown in Fig. 4A, expressions of both p53 and p53 phosphorylation sites, including p-p53 (ser15) and p-p53 (ser37), were upregulated in A2780 cells after exposure to different concentrations of DHM for 48 h and this occurred in a concentration-dependent manner. However, DHM treatment at all tested concentrations did not increase the expression of p-p53 (ser20) and p-p53 (ser46) in A2780 cells and p53 in SKOV3 cells. We then conducted immunofluorescence assay to measure the expression of p53 in the A2780 cells. The fluorescence intensity of p53 was prominently enhanced in the DHM-treated cells (100 μM), while no obvious green fluorescence was observed in the untreated cells (Fig. 4B). Next, we determined the effect of p53 downregulation on the apoptosis of A2780 cells. Notably, knockdown of p53 using p53 siRNA inhibited the expression of p53, leading to a significantly decreased apoptotic rate in response to DHM (Fig. 4C and D).

### Activation of p53 by DHM is responsible for DHM-induced survivin downregulation

To investigate the relationship of p53 and survivin in the regulation of DHM-induced cell apoptosis, we further knocked down the p53 protein with p53 siRNA. The cells were seeded in dishes and transfected with 10 μg/mL siRNA (p53 or scrambled) in 10 μL/dish Lipofectamine^®^ diluted in fresh medium. After 24 h, the cells were exposed to various concentrations of DHM, and the expression of survivin expression was evaluated by Western blotting and apoptosis assays. As expected, the cells transfected with p53 siRNA showed a higher level of survivin than those transfected with scrambled siRNA, indicating the opposite role of p53 and survivin in modulating DHM-induced apoptosis (Fig. 4C). Taken together, our data demonstrated that DHM might downregulate survivin expression via p53 activation, leading to A2780 cell apoptosis.

### DHM sensitizes resistant ovarian cancer cells to paclitaxel and doxorubicin through suppressing survivin expression

**Figure 5:**
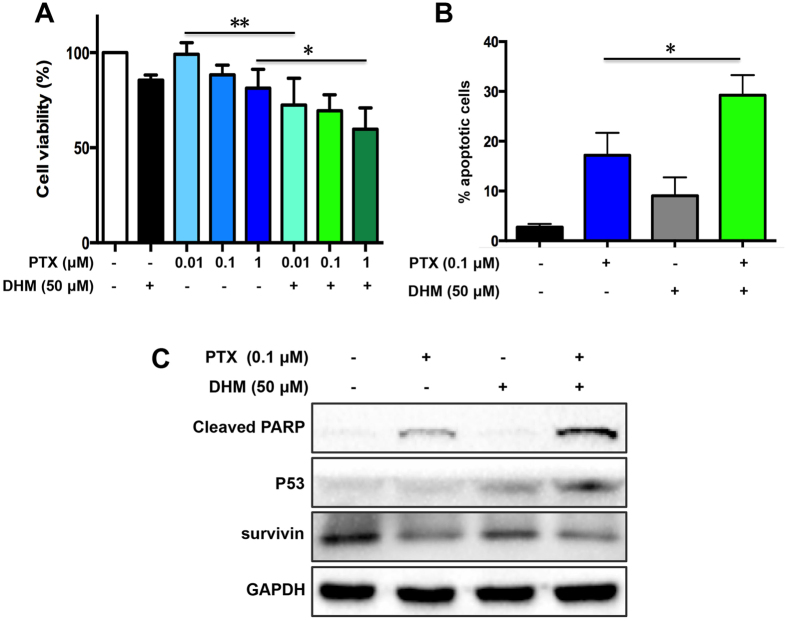
DHM reversed drug resistance in PTX-resistant A2780/PTX cells. (**A**) A2780/PTX cells were exposed to 50 μΜ of DHM, different concentrations of PTX (0.01, 0.1, 1 μΜ), or cotreated with DHM combined with different concentrations of PTX for 48 h. Cell viability was determined by a MTT assay. (**B**) A2780/PTX cells were treated with 50 μΜ of DHM, 0.1 μΜ of PTX, or their combination for 48 h and cell apoptosis was detected by flow cytometry. (**C**) A2780/PTX cells were treated as indicated for 48 h and the expression of cleaved PARP, p53, and survivin was evaluated using Western blot analysis. *p < 0.05, **p < 0.01.

**Figure 6:**
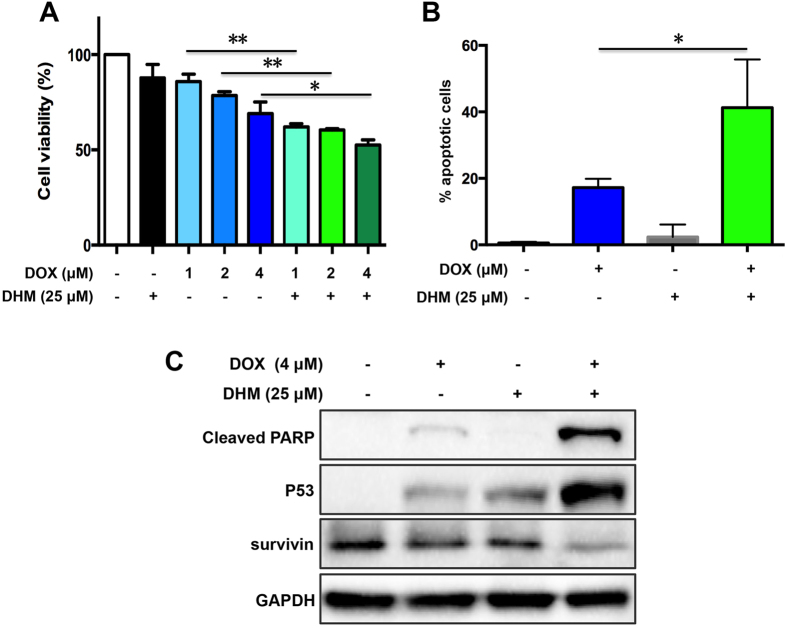
DHM reversed drug resistance in DOX-resistant A2780/DOX cells. (**A**) A2780/DOX cells were exposed to 25 μΜ of DHM, different concentrations of DOX (1, 2, 4 μΜ), or cotreated with DHM combined with different concentrations of DOX for 48 h. Cell viability was determined by a MTT assay. (**B**) A2780/DOX cells were treated with 25 μΜ of DHM, 4 μΜ of DOX, or cotreated with DHM (25 μΜ) and DOX (4 μΜ) for 48 h and cell apoptosis was detected by flow cytometry. (**C**) A2780/DOX cells were treated as indicated for 48 h and the expression of cleaved PARP, p53, and survivin was evaluated using Western blot analysis. *p < 0.05, **p < 0.01.

In retrospective studies, patients with high expression level of survivin show a resistance to chemotherapy and an increased recurrence rate^9^. In tumor tissues, overexpression of survivin for 40-fold renders to cancer cells resistant to chemotherapy. DHM, capable of downergulating the expression of survivin, might serve as a survivin inhibitor and reverse tumor resistance. Currently, paclitaxel (PTX) and doxorubicin (DOX) are two major chemotherapeutic drugs for treatment of ovarian cancer clinically. Thus, we evaluated whether low dose of DHM in combination with PTX and DOX was able to sensitize cancer cells to chemotherapeutic agents. To this end, PTX-resistant A2780/PTX cells were treated with 50 μM of DHM combined with PTX (ranging from 0.01 to 1 μM). Similarly, DOX-resistant A2780/DOX cells were exposed to 25 μM of DHM combined with DOX (ranging from 1 to 4 μM). Cell viability was detected by MTT assay. As shown in Figs 5A and 6A, the cell viability was decreased after treatment of the combination of DHM with either PTX or DOX. Annexin V assay was performed to evaluate their anticancer effects. Figure 5B showed that the apoptotic rate of the PTX group was 17.16%, whereas the combination of DHM and PTX increased the apoptotic rate to 29.25%. Meanwhile, the apoptotic rate in DHM and DOX combination group was 41.27%, 2.4-fold higher than that of DOX group (17.20%) (Fig. 6B). These observations suggested that DHM was able to sensitize A2780 resistant cancer cells to both PTX and DOX.

The expression levels of cleaved PARP, p53 and survivin were visualized by Western blotting. As shown in Figs 5C and 6C, low concentrations of PTX and DOX resulted in low expression levels of cleaved PARP and p53 in A2780 resistant cancer cells, indicating that the resistant cancer cells were relatively unresponsive to these chemotherapeutical agents. However, combination of DHM and PTX or DOX significantly increased the expression of cleaved PARP and p53, suggesting that the combination therapy enhanced the apoptotic effects to some extent. On the contrary, a decreased expression level of survivin was witnessed after combination treatment of DHM and PTX or DOX. This result consistently confirmed that DHM played an important role as a survivin inhibitor and contributed to reducing the expression level of survivin, which was probably modulated by the upregulation of p53, leading to an enhanced pro-apoptotic effects.

## Discussion

Ovarian cancer is the sixth most common cancer among women worldwide. The successful treatment of ovarian cancer has faced several impediments, including a symptomless early state, a high incidence of recurrence and the development of chemoresistance^30^,^31^. The discovery of promising therapeutic agents for ovarian cancer therapy, particularly, to improve the current responses to chemotherapy, remains a key goal for achieving a better outcome. DHM, which is a natural flavonoid that induces negligible side effects in mice, has received wide interest as a potential candidate for cancer therapy^32^. Previous studies have revealed the effects of DHM on cell proliferation, colony formation, cell cycle distribution and cell apoptosis in a plethora of cancer cell lines, such as hepatocellular carcinoma, osteosarcoma, and melanoma cells^31^,^33^,^34^.

Based on the results of our MTT assay, DHM treatment significantly inhibited the proliferation of both A2780 and SKOV3 cells in a concentration- and time-dependent manner but induced no significant cytotoxicity in IOSE80 cells. Obviously, A2780 cells (wild-type p53) were more vulnerable than SKOV3 cells (p53 null) at higher concentrations of DHM (Fig. 1B). For instance, treatment with 200 μM DHM for 24 h decreased the viability of A2780 cells to 70.0% which was significantly lower than that of the SKOV3 cells (84.7%). It was previously documented that the antitumor activity of DHM was at least partially due to the activation of the p53-dependent apoptosis pathway^23^,^32^,^35^. Our results demonstrated that the p53 status might be an important determinant of the sensitivity of ovarian cancer cells to DHM.

Survivin, a nodal protein that belongs to the IAP family, is ubiquitously expressed in various types of cancers. Importantly, it is scarcely detectable in normal cells and tissues, which distinguishes it from other potential targets^36^,^37^,^38^. The downregulation of survivin was reported to be responsible for suppressing the viability and colony formation ability of cancer cells^39^,^40^. Considering its significance in cancer development, survivin has become an important anticancer target^41^,^42^. YM155, a small-molecule survivin suppressant, has shown therapeutic potential against a range of cancers in clinical trials^43^,^44^,^45^. Our results showed that DHM could downregulate the expression of survivin in a concentration-dependent manner. This result was further confirmed by transfecting A2780 cells with pIRES-survivin plasmid, which led to the overexpression of survivin and a lower apoptosis rate induced by DHM compared with the empty vector groups (Fig. 3). These findings supported the fact that the downregulation of survivin is one of the mechanisms involved in DHM-mediated apoptosis.

It was reported that silence of survivin could sensitize ovarian cancer cells to chemotherapeutical agents^46^,^47^. Inhibition of survivin by DHM might facilitate the sensitization to chemotherapeutical agents. Thus, we further evaluated the influence of DHM on conquering drug resistance. Apoptosis assay showed that combination of DHM with PTX or DOX increased the apoptotic rate for 1.7-fold and 2.4-fold on PTX or DOX resistant ovarian cancer cells, respectively, compared to the treatment of PTX or DOX alone. Furthermore, while the treatment of PTX or DOX had little influence on survivin expression levels, and the addition of DHM significantly downregulated the expression of survivin, suggesting that DHM was able to sensitize A2780 resistant cancer cells to both PTX and DOX.

Previous studies have shown that wild-type p53 repressed survivin gene expression transcriptionally by direct binding to survivin promoter and activating p21^29^, and DHM was reported to induce cell apoptosis by activating p53^23^,^32^,^35^. We found that DHM increased the expression of p53 and phosphorylated p53 (ser15) in a concentration-dependent fashion, demonstrating that p53, the “guardian of the genome”, was involved in DHM-triggered apoptosis in A2780 cells. In light of this finding, the association between p53 and survivin has subsequently been investigated. Furthermore, the present study showed changes in the survivin levels after knockdown of p53 (Fig. 4). In addition, while the combination of DHM with PTX or DOX increased the expression level of survivin, the expression level of p53 tended to be lower (Figs 5 and 6). Taken together, it was not surprising that the expression level of survivin showed an increasing trend after DHM treatment, which revealed the biphasic role of p53 and survivin in mediating DHM-triggered apoptosis.

In conclusion, this study demonstrated that DHM may be a promising chemotherapeutic agent for the treatment of ovarian cancer. The suppression of cell proliferation was stimulated by the activation of p53 and the downregulation of survivin. This study also showed a decreased expression level of survivin after DHM exposure, suggesting that DHM may trigger apoptosis though the p53-mediated survivin inhibition. Downregulation of survivin by DHM was also witnessed to sensitize A2780 resistant cancer cells to both PTX and DOX. This finding may shed new light on the direction of an effective strategy for ovarian cancer therapy.

## Methods

### Reagents

A powdered form of DHM, PTX, and DOX were purchased from Dalian Meilun Biology Technology Co., Ltd. (Liaoning, China). DMEM culture medium, fetal bovine serum (FBS), penicillin-streptomycin, 0.25% (w/v) trypsin/EDTA, phosphate-buffered saline (PBS), propidium iodide (PI) and Hoechst 33342 were obtained from Life Technologies (Grand Island, USA). Paraformaldehyde (PFA) and 3-[4,5-dimethyl-2-thiazolyl]-2,5-diphenyl tetrazolium bromide (MTT) were purchased from Sigma Aldrich (St. Louis, MO, USA). Crystal purple was purchased from Beyotime Biotechnology (Jiangsu, China). Primary antibodies against PARP, cleaved PARP, p53, survivin, GADPH and second antibodies were supplied by Cell Signaling Technology (Danvers, MA, USA). All reagent water used was pretreated with a Milli-Q apparatus (Millipore Corporation, Darmstadt, Germany). All other chemicals were of the highest purity commercially available.

### Cell lines and cell culture

Human ovarian cancer A2780 and SKOV3 cells were obtained from Boster Biotech (Wuhan, Hubei, China) and the American type culture collection (ATCC), respectively. Human ovarian surface epithelial IOSE80 cells were obtained from Shanghai Huiying Biotech (Shanghai, China). PTX- and DOX-resistant A2780 cells (A2780/PTX and A2780/DOX) were selected in stepwise increasing concentrations of PTX or DOX as previously described, respectively^27^. Cells were cultured in DMEM medium with 10% (v/v) heat-inactivated FBS and antibiotics (100 U/ml penicillin, 100 μg/mL streptomycin) and maintained at 37 °C in a 5% CO_2_ atmosphere. Cells were passed using trypsin/EDTA, and the medium was changed every other day.

### Cell viability assay

Exponentially growing A2780 and SKOV3 cells were seeded in 96-well plates at a density of 5,000 cells per well in 100 μL of medium and treated with a concentration series of DHM for 24 and 48 h. For drug combination experiments, A2780/PTX were treated with 50 μM of DHM, 0.01 to 1 μM of PTX or cotreated with different concentrations of PTX and DHM for 48 h. A2780/DOX cells were treated with 25 μM of DHM, 1 to 4 μM of PTX or cotreated with different concentrations of PTX and DHM for 48 h. Cell viability was determined with incubation with medium containing 1 mg/mL of MTT for 4 h, followed by dissolving formazan crystals in 100 μL of DMSO. Cell viability was evaluated based on the absorbance at 570 nm by a microplate reader (SpectraMax M5, Molecular Devices, USA). The results were analyzed according to three independent biological replicates.

### Colony formation assay

A2780 cells were seeded in 6-well plates at a density of 50 cells per well. After exposure to 25, 50 and 100 μM DHM for 48 h, the cells were cultured for 2 weeks until colonies formed. Cell colonies were visualized by staining with crystal violet, and colonies with cell counts more than 50 were considered to be surviving colonies.

### Cell cycle analysis

A2780 cells (2.0 × 10^5^ cells) were seeded in 6-well plates following 24 h of starvation. Cells were exposed to 25, 50, and 100 μM of DHM for 24 h, respectively. After trypsinization, cells were harvested and washed twice with PBS, followed by fixation in 70% ethanol at −20 °C overnight. The collected cells were stained with 100 μL of PI stain solution containing 20 μg/mL PI plus 8 μg/mL RNase for 30 min in dark conditions. Sample acquisition was performed using a flow cytometer (BD FACS CantoTM, BD Biosciences, San Jose, USA). The cell distributions in phases of SubG1, G0/G1, S, and G2/M were analyzed using ModFit LT software (version 3.0, Verity, USA). The results were analyzed based on three independent replicates.

### Assessment of apoptosis

Cells were exposed to 25, 50, and 100 μM of DHM for 48 h. After treatment, cell morphology was observed and captured using a microscope (Olympus MVX10, Japan) equipped with a digital camera (ColorView II, So Imaging System, Olympus). Moreover, an Annexin V-FITC/PI apoptosis detection kit was used to detect cell apoptosis. After being treated with various concentrations of DHM for 48 h, A2780 cells were collected by centrifugation, washed twice with cold PBS and suspended in binding buffer. The cells were stained with 5 μL of Annexin-FITC and 5 μL of PI while being protected from light, after which they were analyzed using a flow cytometer (BD Biosciences). For drug combination experiments, A2780/PTX were cotreated with 50 μM of DHM and 0.1 μM of PTX, while A2780/DOX cells were cotreated with 25 μM of DHM and 4 μM of PTX for 48 h, and the same procedures were performed as mentioned above. At least three independent experiments were conducted.

### Caspase 3/7 activity assay

Caspase 3/7 assay was performed using the Caspase3/7-Glo Assay Kit (Promega, Madison, USA). A2780 cells were seeded in white 96-well plates and treated with different concentrations of DHM (25, 50, 100 μM) for 24 h. Then, 100 μL of Caspase 3/7 reagent was added to each well and mixed using a plate shaker. The Caspase 3/7 activity was then determined using a microplate reader (SpectraMax M5). The Caspase 3/7 activity was expressed as a fold of the untreated control treatment. At least three independent experiments were performed.

### Western blotting

After treatment with DHM for 48 h, A2780 cells were lysed with RIPA lysis buffer containing 1% protease inhibitor and 1% phenylmethanesulfonylfluoride (PMSF). For drug combination experiments, A2780/PTX were cotreated with 50 μM of DHM and 0.1 μM of PTX, while A2780/DOX cells were cotreated with 25 μM of DHM and 4 μM of PTX for 48 h, and the same procedures were performed as mentioned above. The samples were incubated in lysis buffer on ice for 20 min and centrifuged at 12,000 g for 20 min, followed by the determination of the protein concentration with a BCA protein assay kit. Equivalent amounts of each sample were subjected to 10% sodium dodecyl sulfate polyacrylamide gel electrophoresis (SDS-PAGE) and transferred onto PVDF membranes. After blocking in a solution of blocking powder for 1 h, the membranes were incubated with primary antibodies (1: 1000) at 4 °C overnight, followed by the corresponding secondary antibodies for 1 h at room temperature. The immunoblots were visualized by enhanced chemiluminescence procedures using a Western blotting detection kit (GE Healthcare Life Sciences). Each immunoblot was repeated in triplicate.

### siRNA transfection

The cells were seeded in dishes (100 mm^2^) and the transfection groups were added to transfection reagents containing 20 μM of siRNA (final concentration) diluted in fresh media without antibiotics. After incubation overnight, the medium was removed, and the cells were treated with various concentrations of DHM 24 h later. Western blots and apoptosis assays were used to evaluate the efficiency of siRNA transfection.

### Plasmid and transfection

An efficient survivin vector was constructed based on the previous method^27^. Briefly, the total mRNA from A2780 cells was used as templates. The forward and reverse primers targeting survivin were 5′-CTTCGAATTCGCCACCATGGGTGCCCCGACGTTGCCCCCTGCCTGG-3′, and 5′-GGGCGGATCCTCAATCCATGGCAGCCAGCTGCTCGATGG-3′, respectively. The purified RT-PCR product was inserted into the pIRES-vector (Clontech, CA, USA) to form the pIRES-survivin plasmid. Using the TurboFect transfection reagent (Thermo Scientific), A2780 cells (2 × 10^5^) were transfected with 3 μg pIRES-vector or pIRES-survivin, followed by incubation with 48 h for subsequent experiments.

### Immunofluorescence

Cells were seeded in 6-well plates for 24 h before being treated with DHM. After treatment for 48 h, cells were fixed in 4% PFA and hydrated with PBS for 1 h, followed by incubation with 5% BSA in 0.2% Triton X-100/PBS for 30 min at room temperature to block non-specific antibodies. The samples were then incubated with primary antibodies overnight at 4 °C. After being rinsed twice with PBS, cells were incubated with a fluorochrome-conjugated secondary antibody diluted in an antibody dilution buffer for 1 h at room temperature in the dark. Cell nuclei were stained with Hoechst 33342. Observations were completed using the In Cell 2000 Analyzer (GE Healthcare Life Sciences). All experiments were conducted in triplicate.

### Statistical analysis

All of the presented data were obtained based on three independent experiments and were expressed as mean ± SD. The results were analyzed by Graphpad Prism 6. Significant differences were assessed using Student’s *t*-test. A significance value of *p* < 0.05 level was considered to be significant in all cases.

## Additional Information

**How to cite this article**: Xu, Y. *et al*. Dihydromyricetin Induces Apoptosis and Reverses Drug Resistance in Ovarian Cancer Cells by p53-mediated Downregulation of Survivin. *Sci. Rep.* **7**, 46060; doi: 10.1038/srep46060 (2017).

**Publisher's note:** Springer Nature remains neutral with regard to jurisdictional claims in published maps and institutional affiliations.

## References

[b1] Siegel R. L., Miller K. D. & Jemal A. Cancer Statistics, 2015. Ca-a Cancer Journal for Clinicians 65, 5–29 (2015). 25559415 10.3322/caac.21254

[b2] Cannistra S. A. Cancer of the ovary. New England Journal of Medicine 351, 2519–2529 (2004). 15590954 10.1056/NEJMra041842

[b3] Ye H., Karim A. A. & Loh X. J. Current treatment options and drug delivery systems as potential therapeutic agents for ovarian cancer: A review. Mater Sci Eng C Mater Biol Appl 45, 609–619 (2014). 25491871 10.1016/j.msec.2014.06.002

[b4] Schwab C. L., English D. P., Roque D. M., Pasternak M. & Santin A. D. Past, present and future targets for immunotherapy in ovarian cancer. Immunotherapy 6, 1279–1293 (2014). 25524384 10.2217/imt.14.90 PMC4312614

[b5] Ffrench B., Gasch C., O’Leary J. J. & Gallagher M. F. Developing ovarian cancer stem cell models: laying the pipeline from discovery to clinical intervention. Mol Cancer 13, 262 (2014). 25495823 10.1186/1476-4598-13-262 PMC4295405

[b6] Dynek J. N. & Vucic D. Antagonists of IAP proteins as cancer therapeutics. Cancer Letters 332, 206–214 (2013). 20685035 10.1016/j.canlet.2010.06.013

[b7] Kanwar J. R., Kamalapuram S. K. & Kanwar R. K. Targeting survivin in cancer: the cell-signalling perspective. Drug Discovery Today 16, 485–494 (2011). 21511051 10.1016/j.drudis.2011.04.001

[b8] Huang Y. . FSH inhibits ovarian cancer cell apoptosis by up-regulating survivin and down-regulating PDCD6 and DR5. Endocrine-Related Cancer 18, 13–26 (2011). 20943720 10.1677/ERC-09-0308

[b9] Altieri D. C. Validating survivin as a cancer therapeutic target. Nature Reviews Cancer 3, 46–54 (2003). 12509766 10.1038/nrc968

[b10] Leuschner F. . Therapeutic siRNA silencing in inflammatory monocytes in mice. Nature Biotechnology 29, 1005–U1073 (2011). 10.1038/nbt.1989 PMC3212614 21983520

[b11] Athanasoula K. C. . Survivin beyond physiology: Orchestration of multistep carcinogenesis and therapeutic potentials. Cancer Letters 347, 175–182 (2014). 24560928 10.1016/j.canlet.2014.02.014

[b12] Jiang B. . Dihydromyricetin ameliorates the oxidative stress response induced by methylglyoxal via the AMPK/GLUT4 signaling pathway in PC12 cells. Brain Res Bull 109, 117–126 (2014). 25451453 10.1016/j.brainresbull.2014.10.010

[b13] Lin B. . A reduction in reactive oxygen species contributes to dihydromyricetin-induced apoptosis in human hepatocellular carcinoma cells. Sci Rep 4, 7041 (2014). 25391369 10.1038/srep07041 PMC4229672

[b14] Meng G. . Attenuating effects of dihydromyricetin on angiotensin II-induced rat cardiomyocyte hypertrophy related to antioxidative activity in a NO-dependent manner. Pharm Biol 1–9 (2014). 10.3109/13880209.2014.948635 25471017

[b15] Shen Y. . Dihydromyricetin as a novel anti-alcohol intoxication medication. J Neurosci 32, 390–401 (2012). 22219299 10.1523/JNEUROSCI.4639-11.2012 PMC3292407

[b16] Zhang K. J., Li Y., Liu W., Gao X. L. & Zhang K. W. Silencing survivin expression inhibits the tumor growth of non-small-cell lung cancer cells *in vitro* and *in vivo*. Molecular Medicine Reports 11, 639–644 (2015). 25333812 10.3892/mmr.2014.2729

[b17] Zhou Y. . Ampelopsin Induces Cell Growth Inhibition and Apoptosis in Breast Cancer Cells through ROS Generation and Endoplasmic Reticulum Stress Pathway. Plos One 9 (2014). 10.1371/journal.pone.0089021 PMC3923868 24551210

[b18] Liu B. . Dihydromyricetin induces mouse hepatoma Hepal6 cell apoptosis via the transforming growth factorbeta pathway. Molecular Medicine Reports 11, 1609–1614 (2015). 25376731 10.3892/mmr.2014.2891 PMC4270327

[b19] Zhang Q. Y. . Dihydromyricetin inhibits migration and invasion of hepatoma cells through regulation of MMP-9 expression. World J Gastroenterol 20, 10082–10093 (2014). 25110435 10.3748/wjg.v20.i29.10082 PMC4123337

[b20] Roy K., Kanwar R. K., Krishnakumar S., Cheung C. H. A. & Kanwar J. R. Competitive inhibition of survivin using a cell-permeable recombinant protein induces cancer-specific apoptosis in colon cancer model. International Journal of Nanomedicine 10, 1019–1043 (2015). 25678789 10.2147/IJN.S73916 PMC4324544

[b21] Zhang Q. . Dihydromyricetin promotes hepatocellular carcinoma regression via a p53 activation-dependent mechanism. Sci Rep 4, 4628 (2014). 24717393 10.1038/srep04628 PMC3982169

[b22] Wu S. . Dihydromyricetin reduced Bcl-2 expression via p53 in human hepatoma HepG2 cells. PLoS One 8, e76886 (2013). 24223706 10.1371/journal.pone.0076886 PMC3817187

[b23] Liu J. . Dihydromyricetin induces apoptosis and inhibits proliferation in hepatocellular carcinoma cells. Oncology Letters 8, 1645–1651 (2014). 25202384 10.3892/ol.2014.2330 PMC4156277

[b24] Zeng G. F. . Dihydromyricetin induces cell cycle arrest and apoptosis in melanoma SK-MEL-28 cells. Oncology Reports 31, 2713–2719 (2014). 24789439 10.3892/or.2014.3160

[b25] Huang H. L., Hu M., Zhao R., Li P. & Li M. Y. Dihydromyricetin suppresses the proliferation of hepatocellular carcinoma cells by inducing G2/M arrest through the Chk1/Chk2/Cdc25C pathway. Oncology Reports 30, 2467–2475 (2013). 24002546 10.3892/or.2013.2705

[b26] Kar R. . Survivin siRNA increases sensitivity of primary cultures of ovarian cancer cells to paclitaxel. Clinical & Translational Oncology 17, 737–742 (2015). 26033427 10.1007/s12094-015-1302-2

[b27] Wang S. P., Wang L., Chen M. W. & Wang Y. T. Gambogic acid sensitizes resistant breast cancer cells to doxorubicin through inhibiting P-glycoprotein and suppressing survivin expression. Chemico-Biological Interactions 235, 76–84 (2015). 25824409 10.1016/j.cbi.2015.03.017

[b28] Nabilsi N. H., Broaddus R. R. & Loose D. S. DNA methylation inhibits p53-mediated survivin repression. Oncogene 28, 2046–2050 (2009). 19363521 10.1038/onc.2009.62

[b29] Raj D., Liu T., Samadashwily G., Li F. Z. & Grossman D. Survivin repression by p53, Rb and E2F2 in normal human melanocytes. Carcinogenesis 29, 194–201 (2008). 17916908 10.1093/carcin/bgm219 PMC2292458

[b30] Garces A. H. I., Dias M. S. F., Paulino E., Ferreira C. G. M. & de Melo A. C. Treatment of ovarian cancer beyond chemotherapy: Are we hitting the target? Cancer Chemotherapy and Pharmacology 75, 221–234 (2015). 25212538 10.1007/s00280-014-2581-y

[b31] Carpi S. . Theranostic Properties of a Survivin-Directed Molecular Beacon in Human Melanoma Cells. Plos One 9 (2014). 10.1371/journal.pone.0114588 PMC4263748 25501971

[b32] Zhang Q. Y. . Dihydromyricetin promotes hepatocellular carcinoma regression via a p53 activation-dependent mechanism. Scientific Reports 4 (2014). 10.1038/srep04628 PMC3982169 24717393

[b33] Duan D. P. . The cyclooxygenase-2 inhibitor NS-398 inhibits proliferation and induces apoptosis in human osteosarcoma cells via downregulation of the survivin pathway. Oncology Reports 28, 1693–1700 (2012). 22922684 10.3892/or.2012.1992

[b34] Shoeneman J. K. . Expression and Function of Survivin in Canine Osteosarcoma. Cancer Research 72, 249–259 (2012). 22068035 10.1158/0008-5472.CAN-11-2315

[b35] Wu S. X. . Dihydromyricetin Reduced Bcl-2 Expression via p53 in Human Hepatoma HepG2 Cells. Plos One 8 (2013). 10.1371/journal.pone.0076886 PMC3817187 24223706

[b36] Mobahat M., Narendran A. & Riabowol K. Survivin as a Preferential Target for Cancer Therapy. International Journal of Molecular Sciences 15, 2494–2516 (2014). 24531137 10.3390/ijms15022494 PMC3958864

[b37] Groner B. & Weiss A. Targeting Survivin in Cancer: Novel Drug Development Approaches. Biodrugs 28, 27–39 (2014). 23955284 10.1007/s40259-013-0058-x PMC3929033

[b38] Altieri D. C. Targeting survivin in cancer. Cancer Letters 332, 225–228 (2013). 22410464 10.1016/j.canlet.2012.03.005 PMC3695618

[b39] Wheatley S. P. & McNeish A. Survivin: A protein with dual roles in mitosis and apoptosis. International Review of Cytology - a Survey of Cell Biology, Vol 247 247, 35-+ (2005). 10.1016/S0074-7696(05)47002-3 16344111

[b40] Church D. N. & Talbot D. C. Survivin in Solid Tumors: Rationale for Development of Inhibitors. Current Oncology Reports 14, 120–128 (2012). 22234703 10.1007/s11912-012-0215-2

[b41] Rodel F. . Survivin as a Prognostic/Predictive Marker and Molecular Target in Cancer Therapy. Current Medicinal Chemistry 19, 3679–3688 (2012). 22680927 10.2174/092986712801661040

[b42] Huang J. C., Lyu H., Wang J. X. & Liu B. L. MicroRNA regulation and therapeutic targeting of survivin in cancer. American Journal of Cancer Research 5, 20–31 (2015). 25628918 PMC4300714

[b43] Rauch A. . Survivin and YM155: How faithful is the liaison? Biochimica Et Biophysica Acta-Reviews on Cancer 1845, 202–220 (2014). 10.1016/j.bbcan.2014.01.003 24440709

[b44] Zhao X. X. . Small Molecule Inhibitor YM155-Mediated Activation of Death Receptor 5 Is Crucial for Chemotherapy-Induced Apoptosis in Pancreatic Carcinoma. Molecular Cancer Therapeutics 14, 80–89 (2015). 25344582 10.1158/1535-7163.MCT-14-0229 PMC4387779

[b45] Kaneko N. . Combination of YM155, a Survivin Suppressant, with Bendamustine and Rituximab: A New Combination Therapy to Treat Relapsed/Refractory Diffuse Large B-cell Lymphoma. Clinical Cancer Research 20, 1814–1822 (2014). 24486595 10.1158/1078-0432.CCR-13-2707

[b46] Chen L. F. . Survivin Status Affects Prognosis and Chemosensitivity in Epithelial Ovarian Cancer. International Journal of Gynecological Cancer 23, 256–263 (2013). 23358177 10.1097/IGC.0b013e31827ad2b8

[b47] Yan X. J. . Triterpenoids as reversal agents for anticancer drug resistance treatment. Drug Discovery Today 19, 482–488 (2014). 23954181 10.1016/j.drudis.2013.07.018

